# 2788. Cefepime or Carbapenem Definitive Therapy Versus Other Antibiotics for Blood Stream Infections Caused by Non-ESBL-Producing *Serratia Marcescens*: A Multicenter Retrospective Cohort Study

**DOI:** 10.1093/ofid/ofad500.2399

**Published:** 2023-11-27

**Authors:** Abdallah Mughrabi, Julian Maamari, Timothy Philips, Afaq Alabbasi, Aislinn Brooks, Rinat Nuriev, Lisa Zenkin, Bertrand Jaber, Claudia Nader

**Affiliations:** St. Elizabeth's Medical Center - Boston University School of Medicine, Boston, Massachusetts; St. Elizabeth's Medical Center - Boston University School of Medicine, Boston, Massachusetts; St. Elizabeth's Medical Center - Boston University School of Medicine, Boston, Massachusetts; St. Elizabeth's Medical Center - Boston University School of Medicine, Boston, Massachusetts; Steward St. Elizabeth's Medical Center of Boston, Rutland, Massachusetts; St. Elizabeth's Medical Center - Boston University School of Medicine, Boston, Massachusetts; St. Elizabeth's Medical Center - Boston University School of Medicine, Boston, Massachusetts; St. Elizabeth's Medical Center - Tufts School of Medicine, Boston, Massachusetts; St. Elizabeth's Medical Center - Tufts School of Medicine, Boston, Massachusetts

## Abstract

**Background:**

*Serratia Marcescens* causes serious infections. Carbapenems were preferred due to fear of inducible AmpC resistance. IDSA guidance now recommends ceftriaxone, and in high-burden cases, cefepime. Evidence on *Serratia* bacteremia treatment is limited. This study compares outcomes of cefepime or carbapenem (CPCT) vs. non-cefepime non-carbapanem containing definitive therapy (NCPCT) in non-ESBL *Serratia* bacteremia.

**Methods:**

We retrospectively reviewed adults (≥18 years) with *Serratia Marcescens* bacteremia hospitalized in seven Massachusetts acute care hospitals (2015 - 2022) **(Figure 1)**. Demographics, clinical course characteristics, and antibiotic therapy choices were obtained. Primary outcome was 30-day mortality, and secondary composite outcome was antimicrobial failure (Definitions in Table 1 and 2). Chi Square and Mann-Whitney U tests were computed using SPSS statistical package.

**Figure 1.**
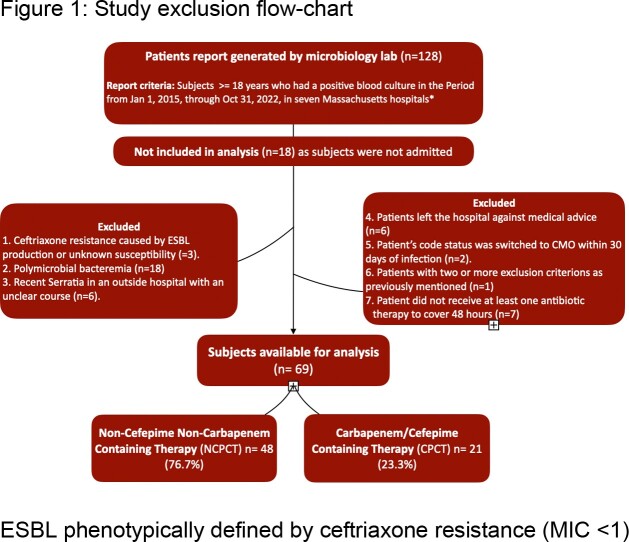
Study exclusion flow-chart. ESBL phenotypically defined by ceftriaxone resistance (MIC <1)

**Results:**

69 out of 128 patients were included in the study. The mean age was 59.23 and 27.53% had IVDU disorder. 21.9 % of patients received CPCT while 78.1% were treated with NCPCT. Notably, more patients in the CPCT group received antibiotics in preceding 3 months, and had more ventilator associated pneumonia (VAP). Other baseline characteristics, bacteremia sources and severity of infection were similar between both groups **(Table 1)**. CPCT patients had longer definitive therapy and more prolonged hospital stay, while more patients in the NCPCT group were switched to oral therapy **(Table 2)**. Neither 30-day mortality rate (8.7% NCPCT vs. 5% CPCT, P= 0.602) nor antimicrobial failure rate (12.5 % NCPCT vs. 23.8% CPCT, P=0.238) was significantly different.

**Table 1.**
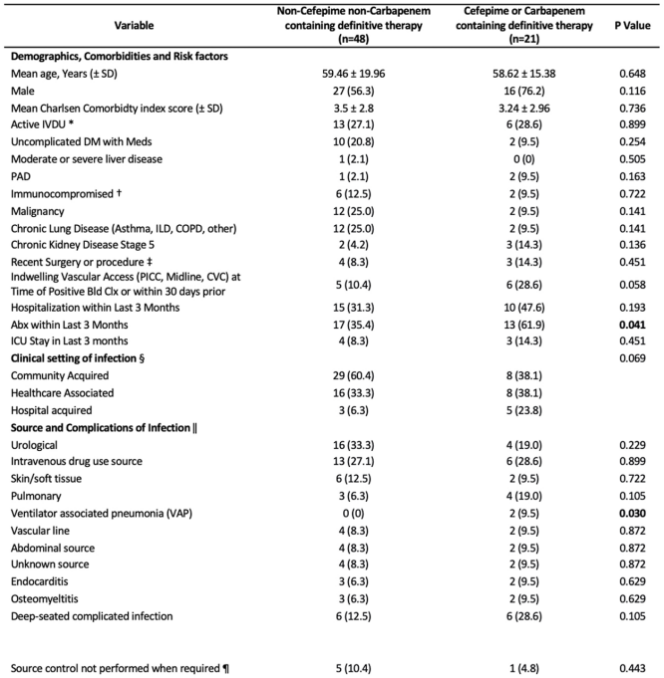
Clinical characteristics and bacteremia sources of patient with Serratia bacteremia treated with a cefepime or carbapenem versus a non-cefepime nor carbapenem therapy regimen. * Immunocompromised patients are those receiving immunosuppressants, chemotherapy, or transplants, having HIV with CD4+ T cell count <200 cells/mm3, neutropenia with ANC < 500 cells/mm3, or corticosteroid use with prednisone or equivalent >10 mg for ≥ 14 days. † Active IVDU is defined as Self-reported or clinical evidence (exam or urine toxicology screen) of active Injection Drug use. ‡ Recent surgery or procedure in the 30 days leading to the positive culture includes urological, Gastroenterological procedure (Endoscopy or Paracentesis), Gastroenterological surgery (Intra-abdominal surgery or peri-anal surgery), Neurosurgical, Respiratory, vascular or Musculoskeletal. § Hospital-acquired bacteremia is diagnosed when a positive blood culture obtained from patients hospitalized for 48 hours or longer. Healthcare-associated bacteremia was defined as a positive blood culture is obtained within 48 hours of hospital admission and the patient fulfills specific pre-defined criteria utilized in prior studies. Community-acquired bacteremia refers to positive a blood culture obtained within 48 hours after hospital admission in patients who do not fit the criteria for healthcare-associated infections. || Urological is defined by pyuria or positive urine cultures. All patients had Pyuria except for 3 patients, instead they had renal calculi and irritative urinary symptoms. Skin and/or soft tissue infection: Per noted clinical examination findings, with some cases supplemented by Serratia growth with Mod or High PMNs from clinical specimens. Ventilator associated pneumonia (VAP): mechanically ventilated for more than 48H before the positive respiratory cultures. Other cases had clinical evidence of infection around port of entry or blood cultures drawn from the line growing Serratia. Intra-abdominal infection included radiological evidence of abscess, diverticulitis, biliary Dilatation, or cholecystitis. All cases were found to have elevated alkaline phosphatase and/or bilirubin. Deep seated complicated infection included Infective endocarditis, septic arthritis, osteomyelitis, or epidural abscess. ¶ Source control included drainage or debridement of deep soft tissue infection, drainage of pleural effusion, drainage of intra-abdominal abscess, resolution of biliary obstruction, cholecystectomy, removal of vascular catheter, removal of indwelling catheter, resolution of urological obstruction and/or replacement of the nephrostomy tube

**Table 2.**
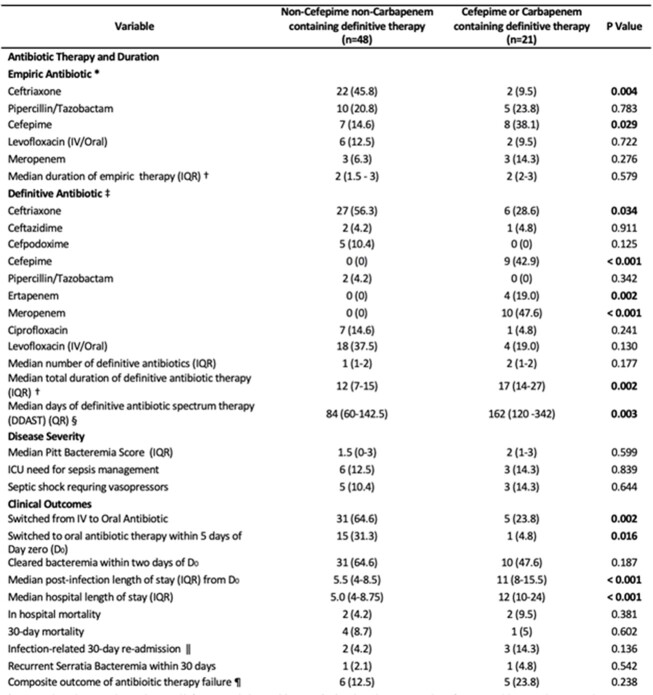
Antibiotic choices and clinical outcomes of patient with Serratia bacteremia treated with a cefepime or carbapenem versus a non-cefepime nor carbapenem therapy regimen. * Empiric antibiotic therapy: Antibiotics administered before antimicrobial susceptibility testing (AST) results, with a minimum 48-hour of uninterrupted duration and appropriate dosing. † Duration of empiric therapy was counted from day one of first empiric therapy until day of AST result. Duration of total definitive antibiotic therapy was counted from day of AST results until last day of definitive antimicrobial therapy. ‡ Definitive antibiotic therapy (i.e., antibiotics administered after the AST results, also with a minimum of 48-hour duration). § Duration of antimicrobial spectrum therapy (DAST) was calculated as follows: In single definitive therapy, spectrum score, per Kakiuchi et al., 2021, was multiplied by the duration in days. In sequential and double antibiotic therapy, the DAST for each antibiotic were summed up. We excluded oral antibiotics prescribed upon discharge from this calculation. || Infection-related re-admission was defined when a patient is readmitted not solely for a procedure or surgery, and they experience fever, worsening leukocytosis, or required escalation of antimicrobial therapy from the definitive therapy of the index admission. ¶ Microbiological failure, microbiological relapse, in- hospital mortality, 30-day mortality, infection-related hospital readmission, or recurrent Serratia bateremia within 30 days. Microbiological failure refers to the growth of Serratia after 48 hours of definitive antibiotic therapy, while microbiological relapse is the regrowth of Serratia after a negative blood culture (Kunz Coyne et al., 2023). Note: Two patients had recurrence of Serratia bacteremia within 30 days, neither were resistant to third generation cephalosporins.

**Conclusion:**

Our study found no significant difference in 30-day mortality or antimicrobial failure between CPCT and NCPCT groups. Furthermore, longer hospital stay was observed in CPCT patients, possibly due to cefepime and meropenem’s less suitability for outpatient antimicrobial therapy (OPAT). Our study is limited by its small size and the overlap in definitive antibiotic therapy. Our findings suggest narrower Beta-lactam therapy may be appropriate which may help early hospital discharge and less days of broad spectrum therapy. Prospective studies are needed to confirm these conclusions and contribute to safe antimicrobial stewardship.

**Disclosures:**

**All Authors**: No reported disclosures

